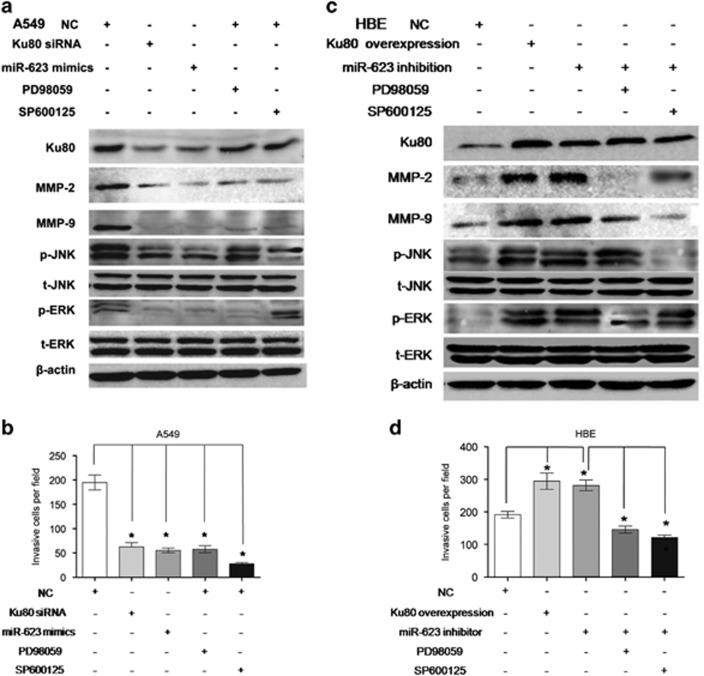# Hsa-miR-623 suppresses tumor progression in human lung adenocarcinoma

**DOI:** 10.1038/cddis.2017.254

**Published:** 2017-05-25

**Authors:** Shuang Wei, Zun-yi Zhang, Sheng-ling Fu, Jun-gang Xie, Xian-sheng Liu, Yong-jian Xu, Jian-ping Zhao, Wei-ning Xiong

**Correction to:** *Cell Death and Disease* (2016) **7**, e2388; doi:10.1038/cddis.2016.260; published online 29 September 2016

Since the publication of this article in *Cell Death and Disease* in 2016, the authors noted an error contained in Figure 7a, in that, the western blot image for MMP9 was incorrect. The correct blot image is now included in the figure given here.

The corrected article appears online together with this corrigendum.

The authors would like to apologize for any inconvenience caused.

## Figures and Tables

**Figure 7:**